# Electroacupuncture Improves M2 Microglia Polarization and Glia Anti-inflammation of Hippocampus in Alzheimer’s Disease

**DOI:** 10.3389/fnins.2021.689629

**Published:** 2021-09-27

**Authors:** Lushuang Xie, Yi Liu, Ning Zhang, Chenyu Li, Aaron F. Sandhu, George Williams, Yan Shen, Hongying Li, Qiaofeng Wu, Shuguang Yu

**Affiliations:** ^1^College of Basic Medicine, Chengdu University of Traditional Chinese Medicine, Chengdu, China; ^2^Institute of Electroacupuncture and Homeostasis Regulation, Chengdu University of Traditional Chinese Medicine, Chengdu, China; ^3^Department of Neurology, Dalian Municipal Central Hospital, Affiliated Hospital of Dalian Medical University, Dalian, China; ^4^Electroacupuncture and Moxibustion College, Chengdu University of Traditional Chinese Medicine, Chengdu, China; ^5^Department of Neurosurgery, Brigham and Women’s Hospital, Harvard Medical School, Boston, MA, United States; ^6^Department of Orthopedic Surgery, Brigham and Women’s Hospital, Harvard Medical School, Boston, MA, United States

**Keywords:** acupuncture, Alzheimer’s disease, microglia, astrocyte, cytokines

## Abstract

**Background:** Alzheimer’s disease (AD) is a neurodegenerative disease characterized by loss of recognition and memory. Neuroinflammation plays pivotal roles in the pathology of AD and affects the progression of the disease. Astrocyte and microglia, as main immune executors in the central nervous system (CNS), participate into the inflammatory response in AD. Glia polarize into different phenotypes during neurodegeneration. Pro-inflammatory glia produce cytokines (IL-1β, TNF-α, and IL-6) resulting into debris aggregates and neurotoxicity. Anti-inflammatory phenotypes produce cytokines (IL-4 and IL-10) to release the inflammation. Electroacupuncture is a useful treatment that has been found to slow the neurodegeneration in animals through experimentation and in humans through clinical trials. The aim of this study was to uncover the mechanisms of glia activation, microglia polarization, and cytokine secretion regulated by electroacupuncture as a treatment for AD.

**Methods:** Twenty male Sprague–Dawley (SD) rats were randomly divided into four groups: Control group (Control), Normal saline group (NS), AD group (AD), and Electroacupuncture group (Acupuncture). The AD and Acupuncture groups were bilaterally injected with Aβ_1__–__42_ into the CA1 field of the hippocampus. The Acupuncture group received electroacupuncture stimulation on the acupoint “Baihui” (GV20) for 6 days per week for a total of 3 weeks. The Morris Water Maze (MWM) was used to evaluate learning and memory capacity. Immunofluorescence was used to stain GFAP and Iba1 of the DG and CA1 in the hippocampus, which, respectively, expressed the activation of astrocyte and microglia. The M1 microglia marker, inducible nitric oxide synthase (iNOS), and M2 marker Arginase 1 (Arg1) were used to analyze the polarization of microglia. The pro-inflammatory cytokines (IL-1β, TNF-α, and IL-6), anti-inflammatory cytokines (IL-4 and IL-10), and pathway-molecules (p65 and Stat6) were tested to analyze the glia inflammatory response by immunofluorescence and polymerase chain reaction (PCR).

**Results:** The MWM results showed that electroacupuncture improves the escape latency time and the swimming distance of AD rats. The number of GFAP and Iba1 cells significantly increased in AD rats, but electroacupuncture decreased the cells. The iNOS-positive cells were significantly increased in AD, and electroacupuncture decreased the positive cells. Electroacupuncture elevated Arg1-positive cells in AD rats. Electroacupuncture decreased the glia pro-inflammatory cytokine expression and increased the anti-inflammatory cytokine expression in AD rats. Furthermore, electroacupuncture inhibited the NF-κB pathway molecule (p65) while raising the Stat6 pathway molecule (Stat6).

**Conclusion:** These results provide evidence that electroacupuncture improves the recognition abilities and memory of AD rats. Electroacupuncture inhibits the activation of glia and polarizes microglia toward the M2 phenotype. Electroacupuncture decreased the pro-inflammatory cytokines (IL-1β, TNF-α, and IL-6) and increased the anti-inflammatory cytokines (IL-4 and IL-10). Furthermore, electroacupuncture affects the immune responses through inhibition of NF-κB pathway but activation of Stat6 pathway.

## Introduction

The most prevalent characteristic of Alzheimer’s disease (AD) is failure of recognition and memory that is accompanied with other clinical features, such as poor judgment or hallucinations. It is extremely difficult to treat the disease and relieve the symptoms (Knopman et al., 2021). Acupuncture, a very useful treatment of traditional Chinese medicine (TCM), has been reported to bring symptom relief or increase the effects of drugs for AD. Acupuncture could improve the scores of Mini Mental State Examination (MMSE). For example, donepezil and acupuncture increased MMSE scores than donepezil alone (MD 2.37, 95% CI 1.53–3.21) (Zhou et al., 2015). We have also shown that electroacupuncture improved Morris Water Maze (MWM) scores in AD model rats (Xie et al., 2018). By using resting-state functional magnetic resonance imaging (rs-fMRI), it was found that acupuncture altered amplitude of low-frequency fluctuations (ALFFs) in some brain areas of AD patients (Zheng et al., 2018). However, the cellular and molecular mechanisms are still unclear in regard to the process of how exactly electroacupuncture treats AD.

The main neuropathologic occurrences of AD are external neuronal β-amyloid plaques (Aβ) and intra-neuronal neurofibrillary tangles (Tau protein) accumulation. The disturbance of immune response has been recognized as core etiology for AD debris aggregation. Immune cells (microglia, macrophage) and non-immune cells (astrocyte, endothelial cell) extremely activate and pro-inflammatory cytokines are secreted during the progression of AD (Knopman et al., 2021). The pathological debris and inflammatory substances activate microglia and astrocytes. The activated microglia transformed into two phenotypes, M1 and M2, in which M1 mainly promotes pro-inflammation factors and neurotoxicity while M2 promotes anti-inflammation factors which lead to protective function (Tang and Le, 2016). It seemed that microglia tended to polarize toward M1 phenotype in AD. Aβ_1__–__42_ triggered the NF-κB pathway that resulted in an increase of M1 microglial markers and a decrease of M2 markers (Xie et al., 2020). The M1 microglia and pro-inflammatory astrocytes produce pro-inflammatory cytokines, such as TNF-α and IL-1β, which enhance the accumulation of debris, oxidative stress, and neurotoxicity (Minter et al., 2016; Rodriguez-Arellano et al., 2016). Some cytokines (IL-1β, IL-6, and TNF-α) increase the risk of AD; however, deletion of these cytokines treats the neurotoxic effects of Aβ plaques and rescues recognition impairments (Minter et al., 2016). The pro-inflammatory factors cause an inflammatory response and neurotoxicity, but anti-inflammatory factors, such as IL-4 and IL-10, extricate the damage and promote tissue repair (Minter et al., 2016; Tang and Le, 2016).

Acupuncture is one of the widely used Chinese traditional therapies and is commonly utilized in other countries. Previous clinical reports and animal experiments supported that Acupuncture improved the symptoms of AD. Acupuncture improves the astrocytic ultrastructure in AD rats, in which the swelling mitochondrial and endoplasmic reticulum had been released (Tang et al., 2019). Manual acupuncture inhibited inflammasome IL-1β production and neuronal apoptosis in SAMP8 mice (Ding et al., 2017). The regulation of the immune response and its constituents are very important mechanisms of acupuncture. We previously found that electroacupuncture balanced the Treg/Th17 ratio through increasing IL-10 and decreasing IL-6 (Sun et al., 2017). Recently, we reported that acupuncture not only activated astrocytes and microglia in the hippocampus of DSS-induced colitis but also treated the mitochondrial dysfunction (Zhang et al., 2020). We also found that electroacupuncture polarized microglia into the M2 phenotype which improved the anti-inflammatory effects in AD rat models (Xie et al., 2018), but it is still not clear if electroacupuncture activates and polarizes glia or affects the cytokine production in different hippocampal areas.

## Results

### The Effect of Electroacupuncture in Memory and Learning of Alzheimer’s Disease

**FIGURE 1:**
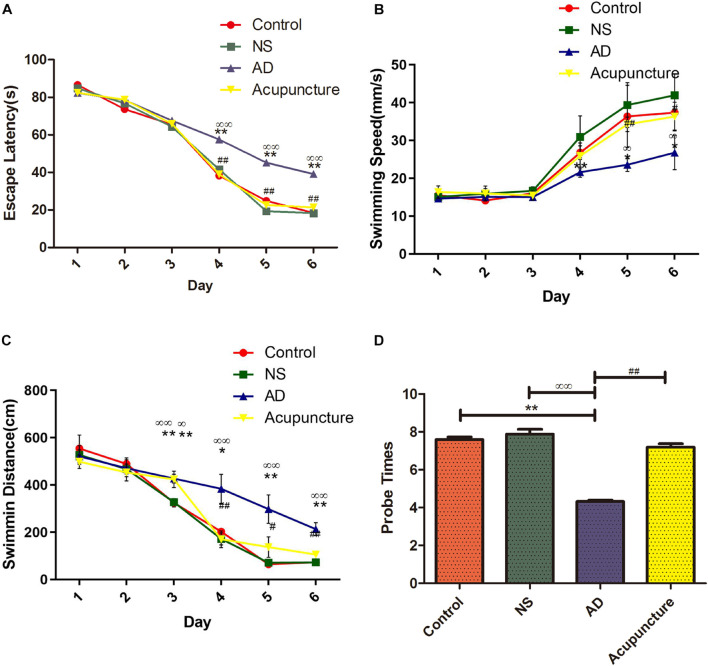
Evaluation of learning and memory capacity in the Morris Water Maze. **(A)** The mean escape latency from star point to the platform. **(B)** The mean swimming speed from the star point to the platform. **(C)** The mean swimming distance. **(D)** Probe times of trails. Compared with the Control group, **p < 0.01; compared with the NS group, ^∞∞^*p* < 0.01; compared with the AD group, ^#^*p* < 0.05, ^##^*p* < 0.01 (*n* = 5).

In order to assess the effects of electroacupuncture on memory and learning, we performed the MWM experiment with rats. Learning and memory have been analyzed *via* the escape latency time and swimming distance. The probe trial has been used to evaluate the memory capacity. The results showed that the AD group (average 39.15 s) was significantly longer compared with Control (average 18.45 s) and NS (average 18.25 s) groups in the escape latency after the 6 days of training, but the mean escape latency of the Acupuncture group (average 21.35 s) was shorter than that of the AD group (Figure 1A). The AD group (average 26.78 mm/s) was significantly lower compared with the Control (average 37.37 mm/s) and NS (average 41.93 mm/s) groups of the mean swimming speed at the sixth day. However, electroacupuncture improved the mean swimming speed upon comparing all groups (Figure 1B). The swimming distance of the Control group (average 72.85 cm) and NS group (average 72.05 cm) was shorter than that of the AD group (average 213.00 cm) on the sixth day, but electroacupuncture decreased the total swimming distance in the Acupuncture group (average 105.60 cm) (Figure 1C). The probe times of the AD group (average 5.0) were less than the Control (average 7.6) and NS (average 7.8). The probe times was improved by electroacupuncture (average 7.2) (Figure 1D).

### Electroacupuncture Released the Glia Activation in Alzheimer’s Disease

**FIGURE 2:**
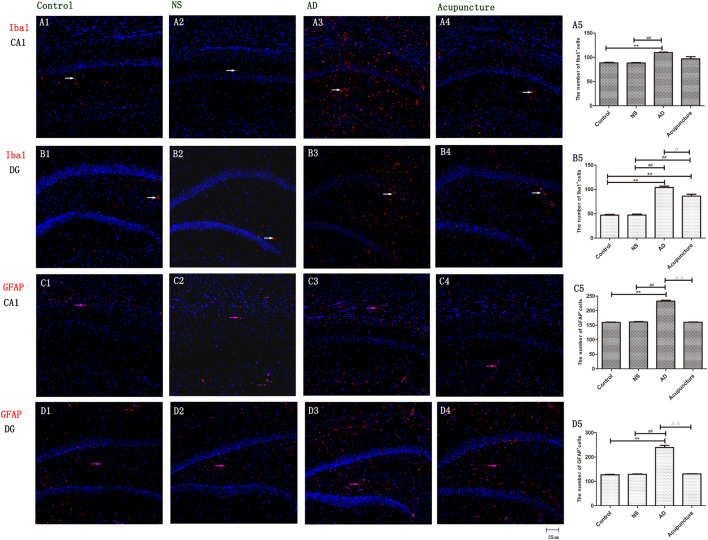
The activation of glia in DG and CA1. **(A1–4)** Microglia of CA1 were stained with anti-Iba1 in four groups. **(B1–4)** Microglia of DG were stained with anti-Iba1. (The white arrow express the red Iba1-positive cells.) **(C1–4)** Astrocyte of CA1 was stained with anti-GFAP. **(D1–4)** Astrocyte of DG was stained with anti-GFAP (the pink arrow express the red GFAP-positive cells). **(A5,B5,C5,D5)** The number of positive stained cells in four groups. Error bars: SE. Compared with Control group, **p < 0.01; compared with NS group, ^##^*p* < 0.01; compared with AD group, ^△^*p* < 0.05, ^△△^*p* < 0.01 (*n* = 5).

The Iba1 is a cytoplasmic helix–loop–helix protein with F-actin binding and actin-cross-linkage. The up-regulation of Iba1 expresses the activation of microglia (Hendrickx et al., 2017). The Iba1^+^ cell levels significantly elevated in the AD group (average 109.8 of CA1 and 104.2 of DG) compared with Control (average 88.4 of CA1 and 47.2 of DG) and NS (average 88.2 of CA1 and 47.4 of DG) groups (*p* < 0.01), but there is no significant difference between the AD and Acupuncture groups (*p* > 0.05) (Figures 2A1–5, B1–5). We used GFAP staining to observe the activation of astrocytes. The GFAP^+^ cells of the Control group (average 159.6 of CA1 and 127.4 of DG) and NS group (average 161.4 of CA1 and 129.4 of DG) are less than the AD group (average 232.6 of CA1 and 239.0 of DG) (*p* < 0.01). The number of positive cells decreased in the Acupuncture group (average 160 of CA1 and 130.6 of DG) (*p* < 0.01) (Figures 2C1–5, D1–5). These results indicate that electroacupuncture inhibited the activation of glia both in the DG and CA1 of the hippocampus.

### Electroacupuncture Improving the M2 Microglia Polarization

**FIGURE 3:**
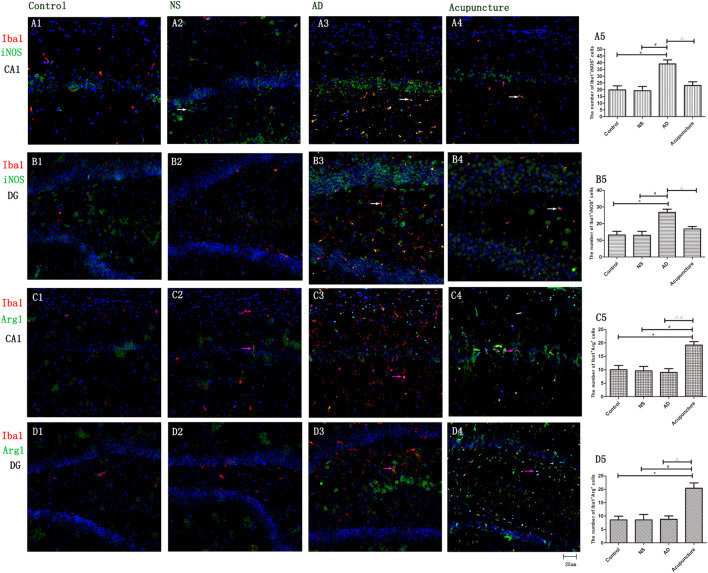
The microglia polarization in different groups. **(A1–4)** Microglia of CA1 was double-stained with anti-iNOS and anti-Iba1 antibody and was observed on fluorescent image. (Red expresses Iba1-positive cells, green expresses iNOS-positive cells, and blue expresses DAPI. The white arrow expresses iNOS^+^Iba1^+^ cells.) **(B1–4)** Microglia of DG was double-stained with anti-iNOS and anti-Iba1 antibody and was observed on fluorescent image (Red expresses Iba1-positive cells, green expresses iNOS-positive cells, and blue expresses DAPI. The white arrow expresses iNOS^+^Iba1^+^ cells.) **(C1–4)** Microglia of CA1 was double-stained with anti-Arg1 and anti-Iba1 antibody and was observed on fluorescent image. (Red expresses Iba1-positive cells, green expresses Arg1-positive cells, and blue expresses DAPI. The pink arrow expresses iNOS^+^Iba1^+^ cells.) **(D1–4)** Microglia of DG was double-stained with anti-Arg1 and anti-Iba1 antibody and was observed on fluorescent image (Red expresses Iba1-positive cells, green expresses Arg1-positive cells, and blue expresses DAPI. The pink arrow expresses iNOS^+^Iba1^+^ cells.) **(A5,B5,C5,D5)** The number of positive stained cells in four groups. Error bars: SE. Compared with Control group, *p < 0.05; compared with NS group, ^#^*p* < 0.05; compared with AD group, ^△^*p* < 0.05, ^△△^*p* < 0.01 (*n* = 5).

To prove that microglia undergo polarization, iNOS was used to mark M1, and Arg1 was used to mark M2. The iNOS is a special enzyme which catalyzes the arginine into citrulline and NO and is usually selected as a functional marker to express the M1 microglia. Arg1 metabolizes arginine into urea and ornithine, then these substances metabolize into hydroxyproline, proline, and polyamine. The Arg1 often is used to identify the M2 microglia (Tang and Le, 2016). The iNOS- and Arg1-positive cells were expressed both in pyramidal cell layer and white matter in the hippocampus (Figure 3). We measured the expression of Iba1^+^iNOS^+^ and Iba1^+^Arg1^+^ cells in CA1 and DG. The double staining results revealed that the number of Iba1^+^iNOS^+^ in AD mice (average 39.0 of CA1 and 26.8 of DG) increased, compared with Control (average 19.8 of CA1 and 13.2 of DG) and NS (average 19.2 of CA1 and 13.0 of DG) (*p* < 0.05) (Figures 3A1–3,5, B1–3,5). Electroacupuncture decreased the Iba1^+^iNOS^+^ cells both in CA1 (average 23.0) and DG (average 16.8) areas (*p* < 0.05) (Figures 3A4–5, B4–5). Electroacupuncture alone increased the Iba1^+^Arg1^+^ cell concentration about twofold in the Acupuncture group (average 15.4 of CA1 and 20.4 of DG), compared with the Control (average 6.4 of CA1 and 8.6 of DG), NS (average 7.2 of CA1 and 8.6 of DG), and AD (average 8.2 of CA1 and 8.8 of DG) groups (*p* < 0.05) (Figures 3C1–5, D1–5). However, there is no significant difference of Iba1^+^Arg1^+^ cells among Control, NS, and model groups (*p* > 0.05).

### Electroacupuncture Reduces the Pro-inflammatory Cytokines of Glia in Alzheimer’s Disease

**FIGURE 4:**
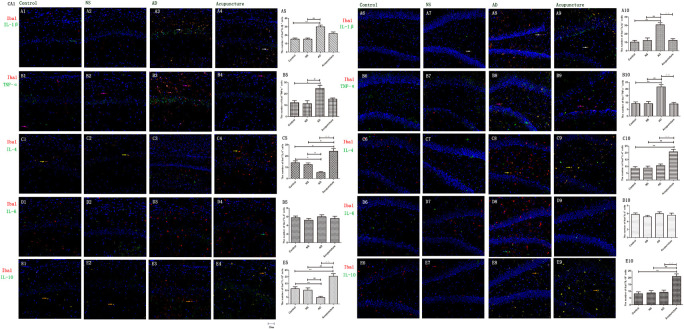
The microglial cytokines in different groups. The microglial cytokines were double-stained with cytokine antibodies and anti-Iba1 antibody. **(A1–4)** Double staining with IL-1β and Iba1 (CA1 area). **(A6–9)** Double staining with IL-1β and Iba1 (DG area). **(B1–4)** TNF-α and Iba1 (CA1 area). **(B6–9)** TNF-α and Iba1 (DG area). **(C1–4)** IL-4 and Iba1 (CA1 area). **(C6–9)** IL-4 and Iba1 (DG area). **(D1–4)** IL-6 and Iba1 (CA1 area). **(D6–9)** IL-6 and Iba1 (DG area). **(E1–4)** IL-10 and Iba1 (CA1 area). **(E6–9)** IL-10 and Iba1 (DG area). (Red expresses Iba1-positive cells, green expresses cytokine-positive cells, and blue expresses DAPI. The white arrow expresses IL-1β^+^Iba1^+^ cells. The pink arrow expresses TNF-α^+^Iba1^+^ cells. The yellow arrow expresses IL-4^+^Iba1^+^ cells. The green arrow expresses IL-6^+^Iba1^+^ cells. The beige arrow expresses IL-10^+^Iba1^+^ cells.) **(A5,B5,C5,D5,E5,A10,B10,C10,D10,E10)** The number of positive double-stained cells in different groups. Error bars: SE. Compared with Control group, *p < 0.05, **p < 0.01; compared with NS group, ^#^*p* < 0.05, ^##^*p* < 0.01; compared with AD group, ^△△^*p* < 0.01 (*n* = 5).

Glia produce pro-inflammatory cytokines, such as IL-1β and TNF-α, which not only enhances the inflammatory response but also elevates neurotoxicity in AD (Grimaldi et al., 2019). Here, we double-stained the different cytokines (IL-1β, TNF-α, and IL-6) and glia markers (GFAP and Iba1). We found that Iba1^+^IL-1β^+^ or Iba1^+^TNF-α^+^ cells were increased in AD (average 30.0 of CA1 and 30.8 of DG for IL-1β, and 24.8 of CA1 and 21.6 of DG for TNF-α), compared to the Control (average 10.4 of CA1 and 10.4 of DG for IL-1β, and 12.2 of CA1 and 9.2 of DG for TNF-α), and NS (average 12.2 of CA1 and 12.2 of DG for IL-1β, and 11.4 of CA1 and 9.2 of DG for TNF-α) groups (*p* < 0.01) (Figures 4A1–3,5–8,10, B1–3,5–8,10). Electroacupuncture decreased the pro-inflammatory factor-positive cells in the DG (average 12.4 for IL-1β and 9.0 for TNF-α) (*p* < 0.01) (Figures 4A9–10, B9–10). But there is no difference between the Acupuncture (average 22.0 for IL-1β and 15.4 for TNF-α) and AD groups in the CA1 (*p* > 0.05) (Figures 4A4–5, B4–5). There is no significant difference of Iba1^+^IL-6^+^ cells among the four groups (*p* > 0.05) (Figures 4D1–10).

**FIGURE 5:**
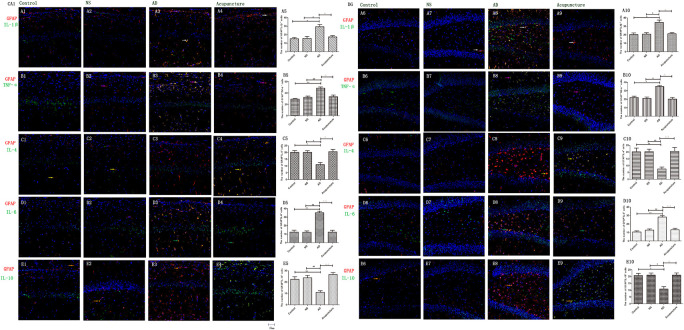
The astrocytic cytokines in different groups. The astrocytic cytokines were double-stained with cytokine antibodies and anti-GFAP antibody. **(A1–4)** Double staining with IL-1β and GFAP (CA1 area). **(A6–9)** Double staining with IL-1β and GFAP (DG area). **(B1–4)** TNF-α and GFAP (CA1 area). **(B6–9)** TNF-α and GFAP (DG area). **(C1–4)** IL-4 and GFAP (CA1 area). **(C6–9)** IL-4 and GFAP (DG area). **(D1–4)** IL-6 and GFAP (CA1 area). **(D6–9)** IL-6 and GFAP (DG area). **(E1–4)** IL-10 and GFAP (CA1 area). **(E6–9)** IL-10 and GFAP (DG area). (Red expresses GFAP-positive cells, green expresses cytokine-positive cells, and blue expresses DAPI. The white arrow expresses IL-1β^+^GFAP^+^ cells. The pink arrow expresses TNF-α^+^GFAP^+^ cells. The yellow arrow expresses IL-4^+^GFAP^+^ cells. The green arrow expresses IL-6^+^ GFAP^+^ cells. The Beige arrow expresses IL-10^+^GFAP^+^ cells.) **(A5,B5,C5,D5,E5,A10,B10,C10,D10,E10)** The number of positive double-stained cells in different groups. Error bars: SE. Compared with Control group, *p < 0.05, **p < 0.01; compared with NS group, ^#^*p* < 0.05, ^##^*p* < 0.01; compared with AD group, ^△^*p* < 0.05, ^△△^*p* < 0.01 (*n* = 5).

**FIGURE 6:**
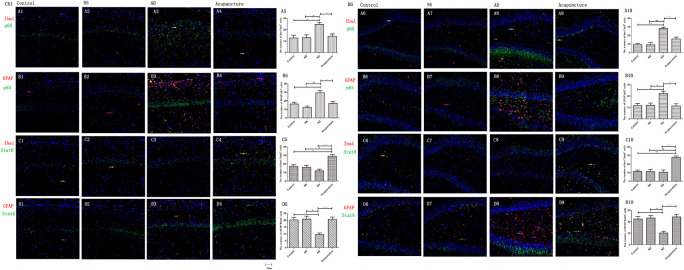
The mRNA expression of cytokines and pathway molecules in hippocampus. **(A–C)** Quantitative data on the relative expression of cytokines mRNA. **(A)** IL-6, **(B)** IL-4, **(C)** IL-10. **(D,E)** Quantitative data on the relative expression of pathway molecules mRNA. **(D)** p65 and **(E)** Stat6 (*n* = 5). Compared with Control group, *p < 0.05, **p < 0.01; compared with NS group, ^##^*p* < 0.01; compared with AD group, ^△△^*p* < 0.01.

The GFAP^+^IL-1β^+^ or GFAP^+^TNF-α^+^ or GFAP^+^IL-6^+^ expression in cells was increased in the AD group (average 29.4 of CA1 and 34.6 of DG for IL-1β, 48.6 of CA1 and 35.0 of DG for TNF-α, and 35.2 of CA1 and 28.2 of DG for IL-6), compared with Control (average 15.2 of CA1 and 20.2 of DG for IL-1β, 29.2 of CA1 and 22.0 of DG for TNF-α, and 12.4 of CA1 and 10.6 of DG for IL-6), and NS (average 15.6 of CA1 and 20.8 of DG for IL-1β, 32.4 of CA1 and 21.0 of DG for TNF-α, and 12.2 of CA1 and 12.8 of DG for IL-6) groups (*p* < 0.05). The number of positive cells decreased due to electroacupuncture (average 17.4 of CA1 and 21.2 of DG for IL-1β, 33.4 of CA1 and 20.2 of DG for TNF-α, and 12.4 of CA1 and 13.2 of DG for IL-6) (*p* < 0.05, *p* < 0.01) (Figures 5A1–10, B1–10, D1–6). The mRNA of IL-6 was elevated in AD compared to Control and NS groups. Electroacupuncture reduced it (Figure 6A).

### Electroacupuncture Elevates the Glia Anti-inflammatory Cytokines in Alzheimer’s Disease

IL-4 and IL-10 are very essential anti-inflammatory cytokines with a crucial role in inhibiting the inflammatory pathological process (da Silva et al., 2015; Bhattarai et al., 2016; Kinney et al., 2018). To quantify the anti-inflammatory cytokines affected by electroacupuncture in AD, we double-stained glia markers and cytokines (IL-4 and IL-10). We found that the expression of Iba1^+^IL-4^+^ or Iba1^+^IL-10^+^ cells in the AD group (average 5.4 for IL-4 and 4.8 for IL-10 in CA1) was two times less than the Control (average 14.0 for IL-4 and 11.2 for IL-10) and NS (average 12.4 for IL-4 and 10.2 for IL-10) groups in CA1 (*p* < 0.05). The positive cells of the Acupuncture group (average 24.2 for IL-4 and 20.4 for IL-10) were more than the other three groups in CA1 (*p* < 0.01) (Figures 4C1–5, E1–5). The expression of Iba1^+^IL-4^+^ or Iba1^+^IL-10^+^ cells significantly increased in the Acupuncture group (average 20.8 for IL-4 and 21.0 for IL-10), compared to the Control (average 8.6 for IL-4 and 8.2 for IL-10), NS (average 8.8 for IL-4 and 8.8 for IL-10), and AD (average 10.6 for IL-4 and 9.2 for IL-10) groups in the DG area of the hippocampus (*p* < 0.01) (Figures 4C6–10, E6–10). The GFAP^+^IL-4^+^ or GFAP^+^IL-10 cells in the AD group (average 11.0 of CA1 and 7.6 of DG for IL-4, and 10.8 of DG and 11.0 of CA1 for IL-10) were about two times less than the Control (average 20.0 of CA1 and 20.2 of DG for IL-4, and 20.8 of DG and 22.4 of CA1 for IL-10), NS (average 20.0 of CA1 and 20.4 of DG for IL-4, and 23.8 of CA1 and 21.0 of DG for IL-10), and Acupuncture (average 20.6 of CA1 and 20.6 of DG for IL-4, and 26.6 of CA1 and 21.0 of DG for IL-10) groups (*p* < 0.05, *p* < 0.01) (Figures 5C1–10, E1–10). The mRNA results reveal that IL-4 and IL-10 decreased in AD mice models compared to the Control and NS mice models (Figures 6B, C). So we can conclude that electroacupuncture increased these anti-inflammatory cytokines expression.

### Electroacupuncture Inhibits the Glia p65 but Increases the Stat6 in Alzheimer’s Disease

**FIGURE 7:**
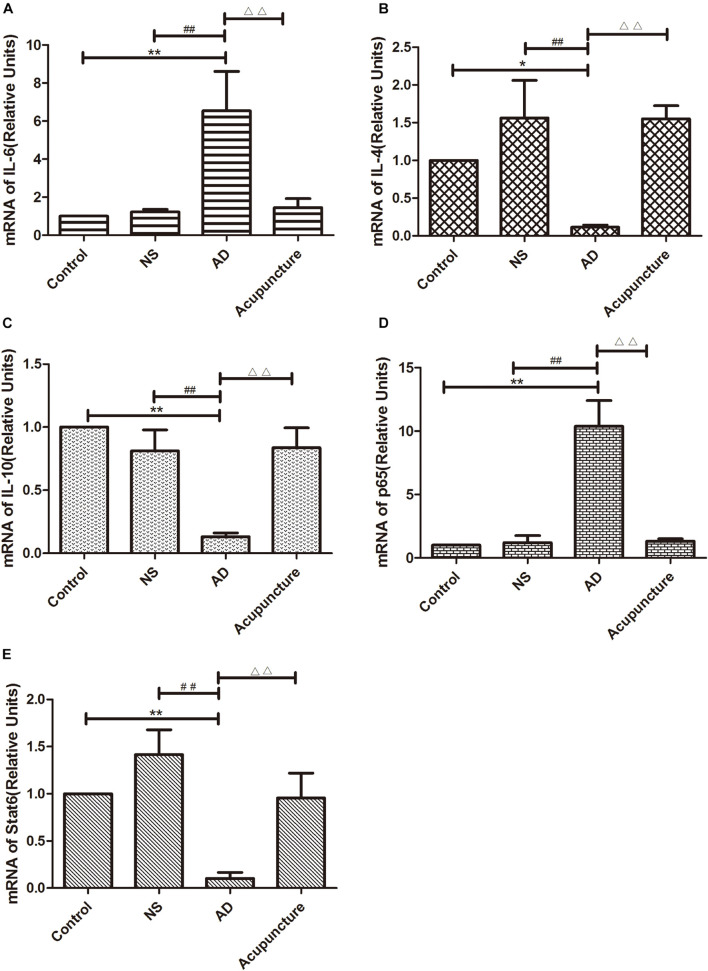
Glia p65 and Stat6 expressed in different groups. **(A, B)** p65 was stained in glia. **(A1–4)** Double staining with p65 and Iba1 (CA1 area). **(A6–9)** p65 and Iba1 (DG area). **(B1–4)** p65 and GFAP (CA1 area). **(B6–9)** p65 and GFAP (DG area). p65 and GFAP (CA1 area). **(C,D)** Stat6 was stained in glia. **(C1–4)** Stat6 and Iba1 (CA1 area). **(C6–9)** Stat6 and Iba1 (DG area). **(D1–4)** Stat6 and GFAP (CA1 area). **(D6–9)** Stat6 and GFAP (DG area). (Red expresses Iba1 or GFAP-positive cells, green expresses p65- or Stat6-positive cells, and blue expresses DAPI. The white arrow expresses p65^+^Iba1^+^ cells. The pink arrow expresses p65^+^GFAP^+^ cells. The yellow arrow expresses Stat6^+^Iba1^+^ cells. The green arrow expresses Stat6^+^GFAP^+^ cells.) **(A5,B5,C5,D5,E5,A10,B10,C10,D10,E10)** The number of positive cells in different groups (*n* = 3). Error bars: SE. Compared with Control group, *p < 0.05, **p < 0.01; compared with NS group, ^#^*p* < 0.05, ^##^*p* < 0.01; compared with AD group, ^△^*p* < 0.05, ^△△^*p* < 0.01 (*n* = 5).

The p65 is the core molecule that contains the activated transcriptional domain. Some pro-inflammatory proteins (such as IL-1β and LPS) induce phosphorylation of IκBs, which are recognized as inhibitory proteins. The p65 translocates into the nucleus and combines its domain after IκBs degradation (Zhong et al., 1998). The number of Iba1^+^p65^+^ cells in the AD group (average 24.8 of CA1 and 28.2 of DG) were twofold more than the Control (average 12.8 of CA1 and 9.4 of DG), NS (average 13.0 of CA1 and 9.2 of DG), and Acupuncture (average 14.4 groups of CA1 and 15.8 of DG) groups (*p* < 0.05, *p* < 0.01) (Figures 7A1–10). The cells expressing GFAP^+^p65^+^ in the AD group (average 29.4 of CA1 and 22.4 of DG) were less than the Control (average 16.4 of CA1 and 11.8 of DG), NS (average 12.4 of CA1 and 12.0 of DG), and Acupuncture groups (average 17.0 of CA1 and 11.4 of DG) (*p* < 0.05) (Figures 7B1–10). The p65 mRNA expression increased in AD groups, compared with the other three groups (*p* < 0.01) (Figure 6D).

Stat6 is the main downstream molecule of IL-4 pathways (Bhattarai et al., 2016). Electroacupuncture increased the Iba1^+^Stat6^+^-positive cells in Acupuncture groups (average 28.0 in DG and 29.0 in CA1), compared to the Control (average 17.2 of CA1 and 11.4 of DG), NS (average 16.0 of CA1 and 11.2 of DG), and AD (average 12.0 of CA1 and 10.4 of DG) groups (*p* < 0.01) (Figures 7C1–10). The GFAP^+^Stat6^+^ cells significantly decreased in the AD groups (average 9.6 in CA1 and 10.2 in DG), compared to the Control (average 20.2 in CA1 and 22.4 in DG), and NS (average 20.8 of CA1 and 23.2 of DG) groups (*p* < 0.05). Surprisingly, electroacupuncture restored the loss of positive cells in the Acupuncture group (average 20.6 in CA1 and 24.2 in DG) (*p* < 0.01) (Figures 7D1–10). The Stat6 mRNA expression decreased in the AD group when compared with the Control and NS groups, but the electroacupuncture increased Stat6 mRNA expression (*p* < 0.01) (Figure 6E).

## Discussion

The activation of microglia and astrocytes are featured in the neurodegeneration, which play very important roles in several functions of the central nervous system (CNS), including inflammation, plasticity, and repair (Knopman et al., 2021). Recently, it was discovered that different types of activated microglia contributed to many physiologies and pathology in neurodegeneration (Tang and Le, 2016). In the present study, we observed that microglia tend to polarize toward the M1 phenotype, that accompanied pro-inflammation in AD rats (Xie et al., 2018). Here, we investigated the microglial polarization in different areas of the hippocampus. Results showed that microglia polarized into an M1 phenotype both in the DG and CA1 areas in the AD group; however, electroacupuncture reversed this effect, which decreased M1 microglia and increased M2 microglia. These results indicated that rebalancing microglia polarization in the hippocampus would be one of the mechanisms of electroacupuncture in AD. The basic function of the DG has been recognized as neurogenesis because it contains neural progenitor cells in adults (Nakashiba et al., 2012). The microglia have a large effect on neurogenesis *via* inflammatory mediation and phagocyted ability (Sierra et al., 2010; Gebara et al., 2013). In the absence of the immune response, the loss of microglia-induced progenitor cells decreased (Gebara et al., 2013), and activated microglia maintain a balance of neurogenesis *via* phagocytosis, the ability of which is affected by the polarization of microglia (Sierra et al., 2010; Tang and Le, 2016). In this study, we found that electroacupuncture tended to improve the microglia polarization into the M2 phenotype and enhance the anti-inflammatory ability in DG area of hippocampus. These results indicated that electroacupuncture would help neurogenesis *via* increasing microglia phagocytosis and inhibiting the pro-inflammation process. The other area of hippocampus, CA1, is usually recognized as an important region that is involved in cognitive processes, especially memory and learning (Rodriguez et al., 2010). The increase of activated microglia was related with memory impairment. It was reported that the number of activated microglia showed a positive relationship with the aggregation of Aβ deposits and neurofibrillary tangles in CA1 (McGuiness et al., 2017). This study supported that electroacupuncture inhibited excessive microglia activation and restored the microglia pro-inflammatory process. This alteration would be one of mechanism of electroacupuncture that is beneficial to neuroprotection and tissue repair. Like microglia, astrocytes are known to have a crucial role in physiological aging and AD progression. The fundamental functions of astrocytes include inflammation, homeostasis, and regeneration (Rodriguez-Arellano et al., 2016; Knopman et al., 2021). Activated astrocytes surround the Aβ plaques and abnormally release neurotransmitters to disturb the normal neuronal activity (Knopman et al., 2021). It was previously recognized that the astrocyte activity of AD patients increased in some areas, especially in the hippocampus, but not in the whole brain (Rodriguez-Arellano et al., 2016). The high density of astrocytes in AD patients mainly accumulated in the CA1/2 regions of the hippocampus (Marlatt et al., 2014). Contrarily, we observed that the activation of astrocytes increased both in CA1 and DG areas. Lian et al. (2016) reported that astrocytic C3 release enhanced Aβ aggregation *via* trigging the microglia C3R, which has been known to be an important crosstalk between glia in the pathology of AD. Thus, inhibiting the excessive activation of astrocytes is one of effective way to slow or delay the progression of the disease. Maintaining homeostasis has widely been recognized as a fundamental mechanism of electroacupuncture (Sun et al., 2017; Xie et al., 2018). We previously reported that acupuncture restores the altercations of Treg and Th17 that triggered the pro-inflammatory response in ulcerative colitis (UC) (Sun et al., 2017). Liang et al. (2016) observed that acupuncture inhibited glia inflammatory response which was the mechanism that relieved neuropathic pain. According our results, we approved that electroacupuncture could inhibit the activation of both microglia and astrocytes to improve the memory loss in AD, while improving the M2 microglia polarization to support the neuroprotection.

The regulation of cytokine is one of fundamental roles of acupuncture, specifically by maintaining the balance of different immune cells, such as T cells and microglia (da Silva et al., 2015; Sun et al., 2017; Xie et al., 2018). In a previous study, we discovered that electroacupuncture decreased the pro-inflammatory factor concentration (IL-1β and TNF-α) in AD animal models (Xie et al., 2018). We further supported that electroacupuncture increased some Treg cytokines (TGF-β, IL-10, and IL-2) while decreasing some Th17 cytokines (IL-6 and IL-17A) *via* the TLR pathway and manipulating the TL17/Treg ratio (Sun et al., 2017). The M2 microglia secreted protective proteins which inhibit neurodegeneration (Tang and Le, 2016). Here, we observed that electroacupuncture expressed anti-inflammatory function in hippocampus. Electroacupuncture increased the concentration of anti-inflammatory factors (IL-4 and IL-10) and decreased the concentration of pro-inflammatory factors (IL-1β, TNF-α, and IL-6) of microglia and astrocytes in the hippocampus, which was associated with the inhibition of glial activation and M2 microglial polarization. IL-4 and IL-10 are the major anti-inflammatory factors that antagonize the inflammatory damage and promoted recovery responses (da Silva et al., 2015; Tang and Le, 2016). The dimers of IL-4 and IL-13 activate the Stat6 pathway *via* combing with IL-4 receptors (Bhattarai et al., 2016). IL-4 and IL-13 are widely accepted as M2 macrophage/microglia polarization stimulators (Bhattarai et al., 2016; Tang and Le, 2016). The IL-4/Stat6 pathway withstands the pro-inflammatory response and improves neurogenesis (Bhattarai et al., 2016). Here, we found electroacupuncture could increase the levels of Stat6 and the anti-inflammatory factors IL-4, both in the astrocyte and the microglia. These results indicated acupuncture would affect IL-4/Stat6 pathway to control the polarization of microglia and inflammatory process of glia in AD. Unlike protective M2 microglia, M1 microglia and activated astrocytes produce some pro-inflammatory cytokines (IL-1β and TNF-α), which enhance the tissue damage and neurotoxicity (Rodriguez-Arellano et al., 2016; Tang and Le, 2016). The NF-κB pathway has been widely recognized as a main inflammatory signal involving into the progression of AD, which was related with cognitive decline (Zhao et al., 2018). Pro-inflammatory cytokines activate the NF-κB pathway, and initiation of the pathway enhances some toxic cytokines. Finally, the inflammatory chain causes neurodegeneration (Tang and Le, 2016). Electroacupuncture inhibited glia activation, decreased pro-inflammatory cytokine, and down-regulated the NF-κB pathway in the hippocampus. These results indicated that NF-κB should be the signaling mechanism that electroacupuncture acts on to control the pro-inflammatory response in AD.

## Conclusion

Electroacupuncture inhibited the activation of glia and improved the M2 microglia polarization in both the CA1 and DG areas of the hippocampus in AD. The anti-inflammatory effects are associated with increasing levels of anti-inflammatory cytokines (IL-4 and IL-10) and decreasing levels of pro-inflammatory cytokines (TNF-α, IL-1β, and IL-6) of glia. The NF-κB and Stat6 pathways might be mechanisms in which the treatment works through.

## Materials and Methods

### Morris Water Maze

The MWM was used to evaluate the learning and memory capacity of the animals on day 5 after Aβ_1__–__42_ injection. The water maze consisted of a flat black metal cylindrical tank (150 cm in diameter and 60 cm in height) that was equipped with a fixed platform (9 cm diameter and 30 cm height) below the surface of the water. A camera recording system was used to record the motion of the animals, accompanied with the MWM analysis software. The swimming pool was divided into four quadrants (I, II, III, and IV) and the platform was located in the four quadrants. The temperature of water was 22 ± 1°C and the indoor environment was room temperature. Before the learning and memory trail, animals were faced with adaptive training for 3 days, in which animals were put on the platform for 10 s to familiarize themselves with the task. For the spatial memory trail, animals were placed in different quadrants of the same pool, and the trail was ended once the animal reached the platform. If the animals did not reach to the platform within 90 s, animals were helped to the platform and the latency data were recorded as 90 s. The spatial memory of the animals was analyzed *via* some data, including the time, swimming distance, and speed from when the animal was put in the water to the time it took for the rat to reach the platform. The time of spatial memory trail was 6 days. On Day 7, a spatial probe trial was conducted *via* the same tank without the platform. The times of crossing the original platform location was recorded within 120 s.

### Animal

Three-month-old Sprague–Dawley (SD) rats were bought from the Sichuan Dashuo Experimental Animal Co., Ltd. [License number: SCXK (chuan) 2015-030]. All experiments were performed in accordance with the guidance of the Institutional Animal Care and Use Committee of Chengdu University of Traditional Chinese Medicine. Rats were individually housed with free access to water and food at a room temperature in ventilated conditions. These rats were performed in adaptive feeding for 1 week. After that, rats were randomly divided into four groups: the Control group, the Normal saline group (NS), the AD group (AD), and the Electroacupuncture group (Acupuncture).

### Aβ_1__–__42_ Injection Induced the Alzheimer’s Disease Rat Model

The surgery was performed as previously described (Li et al., 2014). The AD and electroacupuncture rats were bilaterally injected with Aβ_1__–__42_ into the CA1 field of hippocampus. In brief, rats were anesthetized with intraperitoneal injection of 10% chloral hydrate and fixed on stereotaxic frame. Bilateral injection locations were chosen with the guidance of the rat brain in stereotaxic coordinates (George Paxinos, 2006). The implanted location: AP = 3 mm; L = 2 mm; and V = 4 mm. Each injection contained 2.5 μl Aβ_1__–__42_ (0.01 mg/L) and the NS group had the same volume (0.9%) injected into them.

### Electroacupuncture Treatment

The Acupuncture group got electroacupuncture treatments. The treatments began 3 days after surgery and the days of total treatment was 18. Each treated period was 6 days and 1 no-treatment day between each period. Stainless steel needles (0.24 mm in diameter and 13 mm in length) were inserted into the acupoint “Baihui” (GV20). The “Baihui” locates at the intersection between the sagittal midline of the head and the coronary midpoint of the two ears. The location of GV20 was selected according to Government Channel and Points Standard GB12346-90 of China and “The Veterinary Electroacupuncture of China.” The depth of injection was 2 mm. Needle handles were connected with an electroacupuncture apparatus (Hans-200, China). Stimulating parameters: frequency 20 Hz, current strength 20 mA, voltage 2–4 V, and the treatment duration was 30 min.

### Immunofluorescence Analysis

The rats were killed by intraperitoneal injection (10% chloral hydrated, 1 ml/100 g) and 4% paraformaldehyde (PFA) was used to fix *via* cardiac perfusion. Coronal brain slices (15 μm thick) were prepared and used for staining. Slices were washed in PBS for 5 min, three times, then blotted in 5% goat serum for 1 h at room temperature. Slices were incubated with the primary antibodies overnight at 4°C. Primary antibodies used: mouse anti-Iba1 (1:200 Sigma American), mouse anti-GFAP (1:200 Sigma American), rabbit anti-iNOS, anti-Arg1, anti-IL-1β, anti-TNF-α, anti-IL-4, anti-IL-6, and anti-IL-10 (1:200; Bioss, Beijing, China). After having been washed in PBS three times, these slices were incubated with secondary antibodies at 37°C for 2 h. Secondary antibodies: Alexa Fluor 488-conjugated goat anti-rabbit IgG (1:200; Bioss, Beijing, China), Cy3-conjugated goat ant-mouse IgG (1:200; Bioss, Beijing, China). Then these slices were washed three times again and stained with DAPI (Biyuntian, China) for 5 min. Results were imaged using a 7266-fluorescence microscope (Leica, Japan).

### Quantitative Polymerase Chain Reaction Analysis

These rats were sacrificed by cervical dislocation. The hippocampus tissues were extracted and ground into pieces. The RNA of the whole sample was isolated and extracted using TRIzol (Abcame). The cDNA was extracted by using cDNA Synthesis Kit (Biyuntian, China). Quantitative RT-PCR (Q-PCR) was performed on a polymerase chain reaction (PCR) cycler (Bio-Rad CFX96) utilizing the synthetic primers and SYBR Green (Sangon Biotech, China). Samples were subjected to the following reaction systems: 95°C for 3 min, 95°C for 10 s, renaturation for at 60°C for 30 s, and repeated for 45 cycles. ^–△△^Ct was used to calculate the relative expression of mRNA. The sequences of the primers were as follows: IL-6, forward, 5′-AGAAGACCAGAGCAGATTTT-3′ and reverse, 5′-GAGAAAAGAGTTGTGCAATG-3′; IL-4, forward, 5′-CTTT GAACCAGGTCACAG-3′ and reverse, 5′-CTCGTTCTCCGT GGTGTT-3′; IL-10, forward, 5′-CAGAAATCAAGGAGCAT TTG-3′ and reverse, 5′-CTGCTCCACTGCCTTGCTTT-3′; p65, forward, 5′-CTGTTTCCCCTCATCTTTCCCT-3′ and reverse, 5′-CTGGTCCTGTGTAGCCATTGA-3′; and Stat6, forward, 5′-ATGCTTCCATGCAACTCAGC-3′ and reverse, 5′-GCTCCTGAAAAGATGGCAGT-3′.

### Statistical Assay

Data were expressed as means ± SEM. One-way ANOVA Multiple was used to value the comparisons between different groups. Differences were considered significant at *p* < 0.05.

## Data Availability Statement

The raw data supporting the conclusions of this article will be made available by the authors, without undue reservation.

## Ethics Statement

The animal study was reviewed and approved by Institutional Review Board of Chengdu University of Traditional Chinese Medicine.

## Author Contributions

SY and QW conceived and designed the experiments. LX, YL, NZ, CL, AS, GW, YS, and HL performed the experiments. LX, YL, NZ, CL, and QW analyzed the images and data. SY, QW, LX, AS, and GW wrote the manuscript. LX and YL contributed equally to this work. All authors contributed to the article and approved the submitted version.

## Conflict of Interest

The authors declare that the research was conducted in the absence of any commercial or financial relationships that could be construed as a potential conflict of interest.

## Publisher’s Note

All claims expressed in this article are solely those of the authors and do not necessarily represent those of their affiliated organizations, or those of the publisher, the editors and the reviewers. Any product that may be evaluated in this article, or claim that may be made by its manufacturer, is not guaranteed or endorsed by the publisher.
